# Luminescent Wearable Sensor on Anions from Cotton Fabric Grafted with Cu-In-Zn-S Colloidal Quantum Dots

**DOI:** 10.3390/s26113569

**Published:** 2026-06-04

**Authors:** Xiao Liu, Pengbo Zhu, Hao Ren, Yao Wang, Jun Li, Yan Zhang, Qiao Wang, Soo Wohn Lee, Laurence A. Belfiore, Mikhail Artemyev, Jianguo Tang

**Affiliations:** 1 Institute of Hybrid Materials, National Center of International Research for Hybrid Materials Technology, National Base of International Science & Technology Cooperation, College of Materials Science and Engineering, Qingdao University, Qingdao 266071, China; 19511685310@163.com (X.L.); 17853283053@139.com (P.Z.); zgsdrh@163.com (H.R.); wangyaoqdu@126.com (Y.W.); wwwqqq721@163.com (Q.W.); belfiore@engr.colostate.edu (L.A.B.); 2 Guo Yi Materials (Qingdao) Co., Ltd., Qingdao National University Science Park, 127 HuiZhi Qiao Road, Qingdao High Tech Zone, Qingdao 266071, China; 3 School of Materials Science and Engineering, Shanghai University of Engineering Science, Shanghai 201620, China; jacob_lijun@sues.edu.cn (J.L.); yanzhang@sues.edu.cn (Y.Z.); 4 Department of Energy and Chemical Engineering, Sun Moon University, Asan 31460, Chungnam, Republic of Korea; swlee@sunmoon.ac.kr; 5 Department of Chemical and Biological Engineering, Colorado State University, Fort Collins, CO 80523, USA; 6 Research Institute for Physical Chemical Problems of the Belarusian State University, 220006 Minsk, Belarus; m_artemyev@yahoo.com

**Keywords:** Cu-In-Zn-S quantum dots, fluorescence fabric, optical sensing, anions

## Abstract

**Highlights:**

**What are the main findings?**

- We have successfully fabricated a novel fluorescent cotton fabric (FCF) sensor grafted with hydrophilic MPA-stabilized Cu-In-Zn-S core–shell QDs.
- The FCF sensor demonstrates a PL response when in contact with aqueous solutions containing various anions, including Br^−^, Cl^−^, F^−^, PO_4_^3−^, and OH^−^.

**What are the implications of the main finding?**

- This preparation method is simple, reproducible, and cost-effective, with broad application prospects.

**Abstract:**

Negatively charged molecules and anions are widely present in the natural environment and can pose a threat to aquatic life, affecting their survival and reproduction. As the understanding of the hazards of negatively charged molecules and ions deepens, the need for real-time monitoring governs the development of highly sensitive and convenient sensing materials. Here, highly luminescent Zn-Cu-In-S core–shell colloidal quantum dots were grafted onto cotton fabric to produce a fluorescence cotton fabric (FCF) optical sensor demonstrating photoluminescence response to the presence of several anions in water, such as phosphate (PO_4_^3−^), hydroxide (OH^−^), fluoride (F^−^), chloride (Cl^−^), or bromide (Br^−^). After contact with water solutions containing these anions, PL output from FCF remarkably decreases, with specific functional dependence on the concentration of the selected anions. The fluorescent fabric sensing material is easy to operate, achieving real-time detection of negatively charged groups and showing great potential for application in environmental monitoring.

## 1. Introduction

In recent years, colloidal quantum dots (QDs) have attracted widespread attention due to their unique optical and electronic properties with great application potential, especially in the fields of bioimaging, optoelectronic devices, and sensors [1]. Among them, heavy-metal-free Cu-In-Zn-S QDs have received much attention because of their environmental friendliness, tunable fluorescence emission, and good chemical and photostability [2]. The fluorescence properties of these QDs can be adjusted by changing their size and chemical composition, which endow them with broad prospects in smart clothing, biomedical monitoring, and environmental sensors [3]. The surface of colloidal QDs is usually rich in ligands, which governs their colloidal stability and can provide specific interactions with various objects [4]. Certain substances interacting with the surface ligands affect the charge distribution on the surface of QDs [5]. This can alter the fluorescence or electrochemiluminescence (ECL) response from QDs, thus enabling the optical detection of specific substances. QD-based sensors outperform traditional sensors regarding sensitivity and selectivity and in detecting target molecules at a picomolar level [6]. QD-based sensors are widely used in the detection of biomarkers, cell imaging, and the monitoring of drug release [7], as well as for early cancer diagnostics [8,9,10]. They can detect pollutants in water and air, such as heavy metal ions and volatile organic compounds (VOCs), providing effective tools for environmental protection [11,12]. QD-based sensors can also be used to detect harmful substances in food, such as pesticide residues and pathogens [13,14]. Developing nontoxic and stable QD-based sensors is a key research problem [15,16,17]. In this sense, combining heavy-metal-free Cu-In-Zn-S QDs with fabrics enables the optical detection of specific substances through the fluorescence response of QDs in wearable sensors.

QD-based flexible sensors have shown great potential in wearable health monitoring devices. A high-sensitivity piezoresistive pressure sensor has been developed based on perovskite QDs flexible films [18]. QD-based flexible sensors can monitor the composition changes in sweat generated by human movement in real time, providing support for sports health monitoring [19,20]. Despite the progress made in the laboratory, QD-based flexible sensors still face stability issues in practical applications [11]. For example, the chemical and photostability of QDs and their photoluminescence response may decrease during long-term use [8]. Currently, the large-scale production of QD-based flexible sensors is relatively complex and costly [13].

To prepare a cost-effective and environmentally stable and friendly QD-based flexible fluorescence sensing material, we utilized Cu-In-Zh-S core–shell QDs (CIZS) grafted onto the surface of cotton fibers through a dip-coating and self-assembly process [21]. The fluorescence response from the as-prepared fluorescent cotton fabric (FCF) to several anions (PO_4_^3−^, OH^−^, F^−^, Cl^−^, and Br^−^) of different concentrations in aqueous solutions was tested using a microspectrophotometer.

## 2. Materials and Methods

### 2.1. Chemicals and Materials

Copper(I) iodide (CuI, 99.99%), indium acetate (99.99%), 1-octadecene (ODE >90.0%), oleylamine (Oam, AladdinShanghai, China,90%), sulfur (S, 99.99%), zinc oxide (ZnO, Aladdin, 99.99%), 2-ethylhexanoic acid (Aladdin, >99.0%), 1-dodecanethiol (DDT, Aladdin, 98%), isopropanol (Aladdin, >99%), methanol (Aladdin, >99%), trichloroethylene (Aladdin, 99%), sodium hydroxide (99.99%), 3-mercaptopropionic acid (99%), anhydrous ethanol, sodium bromide (99.99%), sodium phosphate (99.99%), sodium chloride (99%), sodium chloride (99.99%), distilled water, pentaerythritol (99%), 3-Chloro-2-hydroxypropyltrimethylammonium chloride (65 wt. % in H2O), chitosan (≥95%), and analytical-grade JFC penetrant were purchased from Guangzhou Runxin Chemical Co., Ltd. (Guangzhou, China). The cotton fabric was purchased from Dongguan Shuangyu Textile Co., Ltd. (Dongguan, China). All water used was deionized. All chemicals mentioned above were of analytical grade.

### 2.2. Synthesis of CIZS QDs

In a three-necked flask, 0.234 g of indium acetate and 0.0128 g of sulfur powder were first dissolved in a mixture of 15 mL of 1-octadecene (ODE) and 4 mL of oleylamine (Oam), and then heated to 90 °C. Additionally, 0.0153 g of CuI was dissolved in 2 mL of 1-dodecanethiol (DDT) to prepare the Cu^+^ precursor solution. In another test tube, 0.15 g of ZnO was mixed with 1 mL of 2-ethylhexanoic acid and 2 mL of 1-octadecene (ODE), and then heated with a Bunsen burner until ZnO had completely dissolved. The reaction mixture was kept under vacuum at 90 °C for 3 min and then purged with nitrogen and heated up to 120 °C. The Cu^+^ precursor solution was injected into the three-necked flask under vigorous stirring, followed by heating up to 160 °C and maintaining the reaction mixture at this temperature for 3 min. Then, the mixture was cooled to 100 °C, the Zn^2+^ precursor solution injected, and the mixture stirred for 3 min. The temperature was then raised to 240 °C and maintained for 30 min to obtain CIZS QDs.

To increase the chemical and photostability of the CIZS QDs, an additional ZnS shell was overgrown atop them. Here, 0.15 g of ZnO was dissolved in a mixture of 2-ethylhexanoic acid (1 mL) and triethylene glycol dimethyl ether (2 mL), and kept at 100 °C in an oil bath. In parallel, 0.1 g of thiourea was slowly heated in 5 mL of triethylene glycol dimethyl ether and maintained at 100 °C. A reaction mixture with CIZS QDs was cooled to 120 °C, and then 1 mL of oleic acid injected to preserve the colloidal stability of the QDs in the reaction environment. The temperature was then raised to 180 °C, and at this point, the mixed solutions of Zn and sulfur precursors were injected into the reaction mixture using a syringe and stirred for 30 min to complete ZnS shell formation. All manipulations were performed in a nitrogen atmosphere. The reaction mixture was cooled down to room temperature, and the QDs were phase-separated via deposition with isopropanol and centrifugation with additional purification out of organic residuals using the repeated redispersion–centrifugation procedure. Finally, the CIZS QDs were dried under vacuum and stored in the dark in a powder form.

### 2.3. Ligand Exchange Procedure

During this stage, 300 mg of CIZS powder was dissolved in 10 mL of dichloromethane to obtain 0.25 M QDs solution. Next, 160 µL of 3-mercaptopropionic acid (MPA) was added into 1 mL of methanol and 230 µL of 30% sodium hydroxide solution added dropwise to neutralize MPA. The CIZS QD solution was then mixed with sodium 3-mercaptopropionate in methanol and thoroughly stirred at 800 rpm for 60 min to ensure complete ligand exchange with the MPA. An excess of isopropanol solution was added to the mixture, which was then centrifuged at 10,000× *g* rpm for 10 min to separate the insoluble part containing mostly CIZS QDs capped with MPA ligand shell. After centrifugation, the supernatant was removed and 5 mL of methanol added to the deposit, followed by another 5 mL of isopropanol, and the mixture was shaken and centrifuged again. This washing and centrifugation process was repeated three times to purify the QDs of organic residuals.

### 2.4. Preparation of Fluorescent Cotton Fabric

#### 2.4.1. Pre-Treatment of Cotton Fabric

A desizing solution was composed of 10 g/L NaOH aqueous solution and 1 g/L JFC solution (JFC solution is a xylene-free mixture of ethanol, isopropanol, and long-chain hydrocarbons). The cotton fabric was immersed into a desizing solution (bath ratio: 1:50), and then heated to boiling point and maintained for 30 min. The cotton fabric was removed, washed three times with warm water, and finally dried.

#### 2.4.2. Modification of Cotton Fabric

A modification solution was prepared with 60 g/L of 3-Chloro-2-hydroxypropyltrimethylammonium chloride (CHPATC); after heating to 60–80 °C, clean cotton fabric samples (5 cm × 10 cm) were treated in this solution (bath ratio: 1:20) for 10 min. Then, NaOH (15 g/L) was added to the solution and the reaction continued for 40 min. Finally, the cotton fabric was removed and washed with cold water until the pH reached 7.

Two grams of chitosan was dissolved in 2 L of 0.2 M acetic acid–sodium acetate (HAc–NaAc) buffer solution with pH = 4.74. Two to four drops of JFC penetrant were added to the solution, and cotton fabric was immersed into the solution and kept in an oil bath at 70 °C for 1.5 h. The fabric was then removed, dried, and baked in an oven at 180 °C for 2 min to obtain chitosan-modified cotton fabric.

#### 2.4.3. Grafting of QDs onto Cotton Fabric

Thirty milliliters of the CIZS QD solution was placed into a beaker. Two to four drops of JFC penetrant were added to the solution and the mixture kept at 70 °C for 3 h. After completing the procedure, the cotton fabric was removed from the beaker and passed through a pneumatic lab-sized colander at a pressure of 0.2 MPa and speed of 3 m/min to flatten the cotton fabric. Finally, the cotton fabric was dried at 80 °C for 10 min and baked at 180 °C for 3 min to obtain FCF grafted with CIZS QDs.

### 2.5. Characterization

The morphology of CIZS QDs was examined using a transmission electron microscope (JEM-2100). The particle size and zeta potential were measured using a Malvern Zetasizer Nano ZS90 DLS device. Fluorescence properties were analyzed using an Edinburgh FLS1000 steady-state/transient spectrofluorometer equipped with a 450 W xenon lamp as the excitation source and PMT-900 detector with a bandwidth of 0.5 nm. Fluorescence lifetime analysis was conducted using a microsecond excitation pulse light source and PMT-900 detector. The chemical composition of the samples was characterized and analyzed using a Fourier-transform infrared spectrometer, with a scanning range of 4000–500 cm^−1^ and a precision of 0.01 cm^−1^. For scanning electron microscopy and X-ray photoelectron spectroscopy (XPS), we used a JSM7800F (JEOL Ltd., Tokyo, Japan) and an ESCALAB 250Xi+ (Thermo Fisher, Waltham, MA, USA) device, respectively. The analysis chamber was maintained at a vacuum of 4 × 10^−9^ mbar, with an excitation source of Al Kα/α rays (hν = 1486.6 eV), an operating voltage of 14.6 kV, and a filament current of 13.5 mA. A signal accumulation was performed over 20 cycles. A charge calibration was conducted using an energy of 20 eV, a step size of 0.1 eV, and a binding energy of C 1s = 284.8 eV as the energy standard. PL and UV absorption spectra of FCF were registered using a full-spectrum microspectrophotometer (CRAIC 20/30 PV).

### 2.6. FCF Sensing Test on Anions

An appropriate amount of sodium bromide was dissolved in deionized water to obtain 10 mL of 0.1 mol/L solution. The preparation methods for sodium phosphate, sodium chloride, sodium hydroxide, and sodium fluoride were the same as that for sodium bromide. We employed 0.1 mol/L solutions of the aforementioned anions, but aliquots of different volumes were used for each experiment. FCF was cut into 40 fresh 1 cm × 1 cm pieces and labeled sequentially as a1–a8, b1–b8, c1–c8, d1–d8, and e1–e8; the PL intensity of each piece was first recorded as F_0_. A pipette was then used to dispense different volumes of sodium phosphate solution onto the a-series, sodium bromide onto the b-series, sodium chloride onto the c-series, sodium hydroxide onto the d-series, and sodium fluoride onto the e-series pieces. After drying, the PL response was tested using a full-spectrum microspectrophotometer. To ensure measurement reproducibility and minimize positional variation, a fixed sampling area of 2 × 2 mm^2^ was defined using a custom-machined aperture mask placed in direct contact with the fabric surface. The FCF samples were mounted on a translation stage with X-Y micrometer positioning screws, allowing precise relocation of the measurement spot. For each sample, the same spatial coordinates were used for all sequential measurements (before and after anion exposure). Between measurements, the sample was removed from the holder for solution treatment and then repositioned using the recorded coordinates, with a repositioning accuracy of ±0.1 mm. The PL signal from the original FCF was set as F_o_, and FCF exposed to anions in aqueous solution at different concentration as F. The plotted ratio F_o_/F against concentration of anions was used to determine PL quenching characteristics as the sensing parameter.

## 3. Results and Discussion

### 3.1. Characterization of CuInZnS QDs Before and After Ligand Exchange

**Figure 1:**
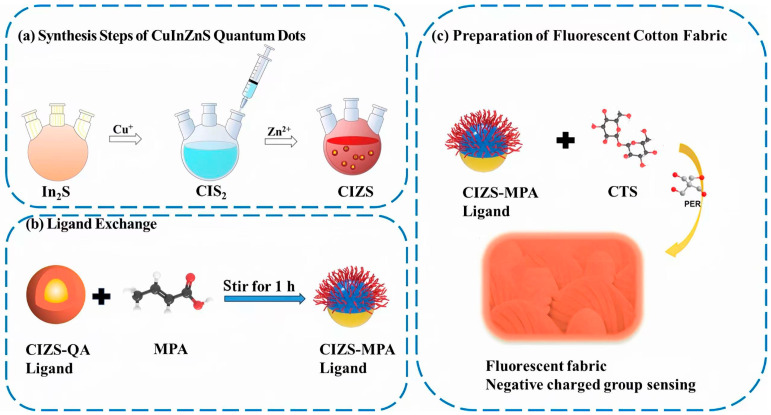
(**a**) Preparation steps for CuInZnS QDs, (**b**) ligand exchange procedure, and (**c**) preparation of FCF.

Figure 1 schematically shows the FCF preparation steps starting from the hot-injection synthesis of hydrophobic CIZS QDs (Panel a), the ligand exchange procedure with hydrophilic MPA molecules (Panel b), and impregnation of the cotton fabric with hydrophilic CIZS QDs (Panel c).

**Figure 2:**
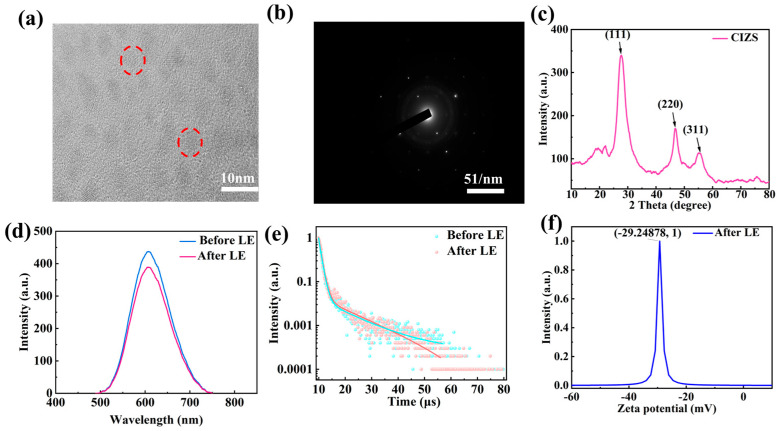
Characterization of CIZS QDs: TEM image (**a**), electron diffraction pattern (**b**), XRD spectrum (**c**), PL spectra of CIZS QDs before and after ligand exchange (**d**), PL decay curves for CIZS before and after ligand exchange, λex = 420 nm (**e**), zeta potential distribution for CIZS QDs in water after ligand exchange (**f**).

Figure 2a shows a TEM image of the as-synthesized CIZS QDs. The nanoparticles are uniformly sized with an average diameter of approximately 8 nm and slightly elongated in shape with a good crystallinity because of clearly visible lattice fringes.

The electron diffraction pattern in Figure 2b confirms the good crystallinity of the QDs, with the lattice spacing of 0.311 nm and 0.167 nm corresponding to (111) and (311) crystalline planes [22].

Figure 2c shows an XRD pattern of the CIZS QDs in a dry powder form. The main diffraction peaks are (111), (220), and (311), which are consistent with the literature [22]. By analyzing the positions of the characteristic peaks in the XRD pattern and using the Scherrer formula [23], the average size of the CIZS QDs was determined to be about 8 nm, which is consistent with the corresponding TEM data.

Figure 2d shows PL spectra of colloidal QDs before (in dichloromethane) and after (in water) the ligand exchange with MPA. The ligand exchange does not remarkably affect the contour and the spectral position of PL band, but the relative intensity decreases.

The PL decay curves in Figure 2e demonstrate that after the ligand exchange, the PL decay time slightly shortens from 9.5 to 5.9 µs due to partial QD PL quenching by surface traps or water molecules. 

Figure 2f shows that CIZS QDs in water possess negative zeta potential (−29 mV) due to the deprotonation of MPA carboxyl groups in the QD surface ligand layer.

### 3.2. FCF Characterization

**Figure 3:**
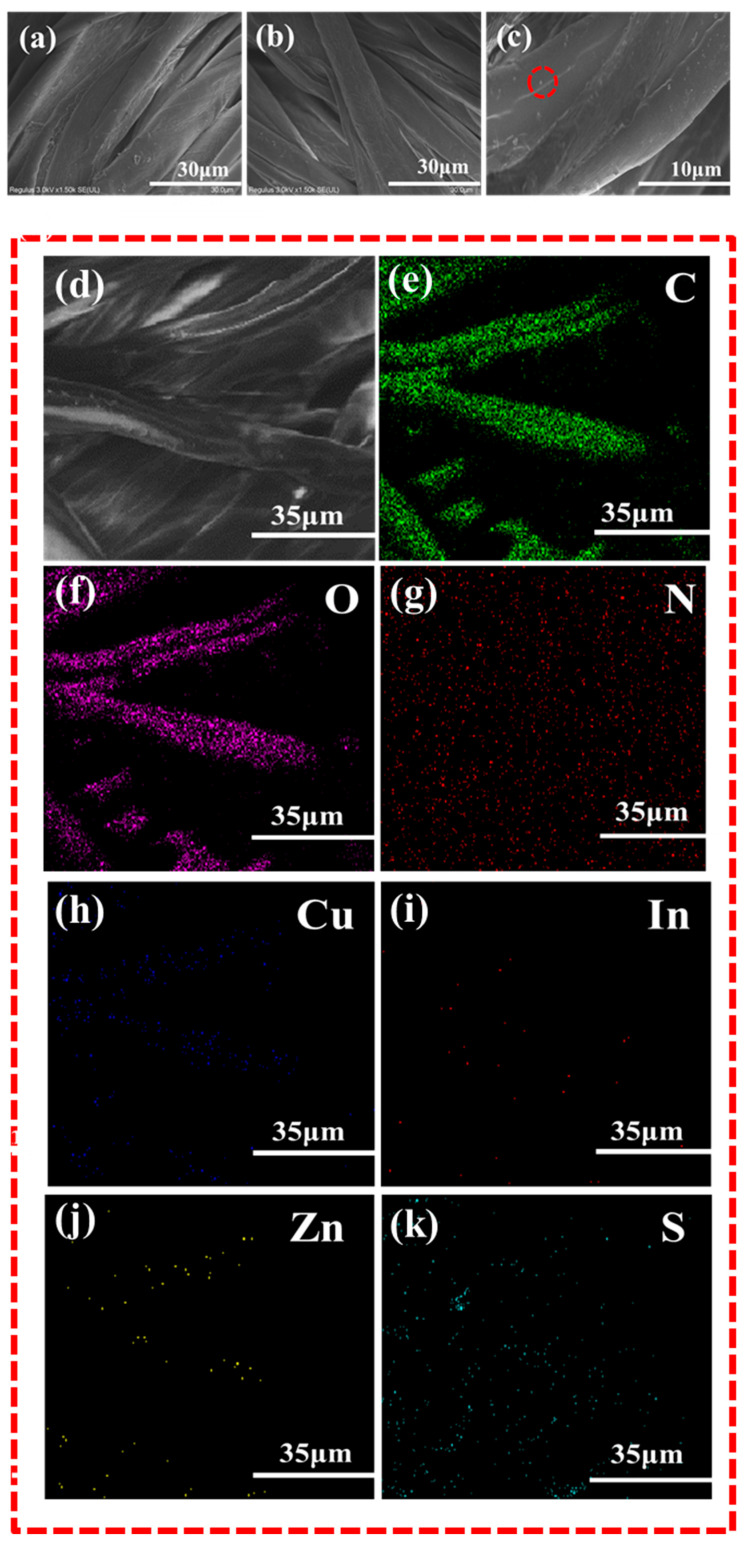
SEM images of nonmodified (**a**) and CHPTAC-modified (**b**) cotton fabric. (**c**) Higher magnification SEM image of FCF sample grafted with CIZS QDs. (**d**) SEM image and elemental mapping for C (**e**), O (**f**), N (**g**), Cu (**h**), In (**i**), Zn (**j**), and S (**k**) atoms of FCF sample.

In an alkaline environment, (3-chloro-2-hydroxypropyl)trimethylammonium chloride (CHPTAC) can undergo a nucleophilic substitution reaction with cellulose [24], thereby cationically modifying the cellulose. The morphology of the cotton fiber surface can be clearly observed through the scanning electron microscopy imaging. Figure 3a shows an SEM image of untreated cotton fabric, while Figure 3b shows an SEM image of the cotton fabric after cationic modification with CHPTAC. The surface of the cationically modified cotton fibers is smooth and remains essentially unchanged, indicating that the surface morphology of the fibers was not damaged during the CHPTAC treatment. Figure 3c shows an SEM image of cotton fabric grafted with CIZS quantum dots, where several spherical nanoparticles can be clearly detected on the surface of cotton fibers. Figure 3e–k shows elemental maps of the FCF sample shown in Figure 3d for carbon, oxygen, copper, indium, zinc, and sulfur atoms. The spatial distribution for Cu and Zn atoms coincide with the O and C distribution of a cotton matrix, indicating that CIZS QDs are uniformly grafted onto the surface of the cotton fibers.

**Figure 4:**
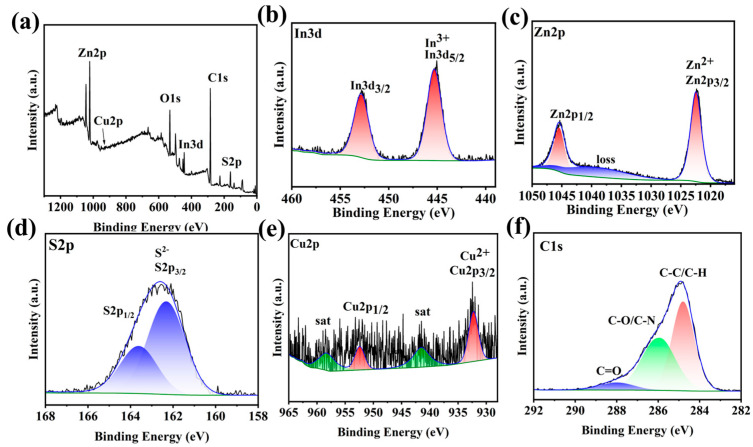
Wide-range (**a**) and high-resolution XPS spectra of FCF sample at In3d (**b**), Zn2p (**c**), S2p (**d**), Cu2p (**e**), and C1s (**f**) binding energy ranges.

The data from the XPS analysis of the FCF sample are shown in Figure 4. In the C1s spectrum, the main peak at 284.80 eV corresponds to the C-C/C-H bonds in the cellulose of the cotton fabric [25,26], serving as the baseline carbon signal. The peak at 285.93 eV is likely associated with C–O (ether) bonds or minor nitrogen-containing functional groups in the cellulose [27]. The peak at 288 eV corresponds to C=O bonds, related to the oxidation products of cellulose in the cotton fabric [28].

In the S2p spectrum, the S2p3/2 peak at 162.32 eV indicates that sulfur exists in the form of S^2−^, consistent with the sulfur state in CIZS QDs [29]. In the In3d spectrum, the In3d5/2 peak at 445.30 eV corresponds to In^3+^, the typical oxidation state of indium in CIZS QDs [30]. In the Zn2p spectrum, the Zn2p3/2 peak at 1022.46 eV indicates that zinc exists as Zn^2+^ [31], while a weak Cu2p3/2 peak at 932.27 eV corresponds instead to Cu^2+^ [32].

**Figure 5:**
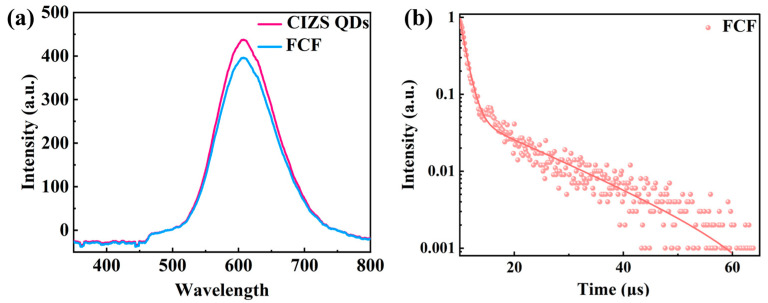
PL spectra of CIZS QD colloids and FCF (**a**) and PL decay curve for FCF (**b**); λex = 420 nm.

Figure 5 compares the PL properties of CIZS QD colloids and FCF. As can be seen from Figure 5a, the emission peak of the QD colloids at ca. 610 nm is almost identical to that of FCF. Figure 5b additionally shows that the PL decay curve for FCF possesses the same characteristics as for the QD colloids in Figure 2e, with an average decay time of about 5.9 microseconds.

### 3.3. Testing FCF Sensor on Anions

**Figure 6:**
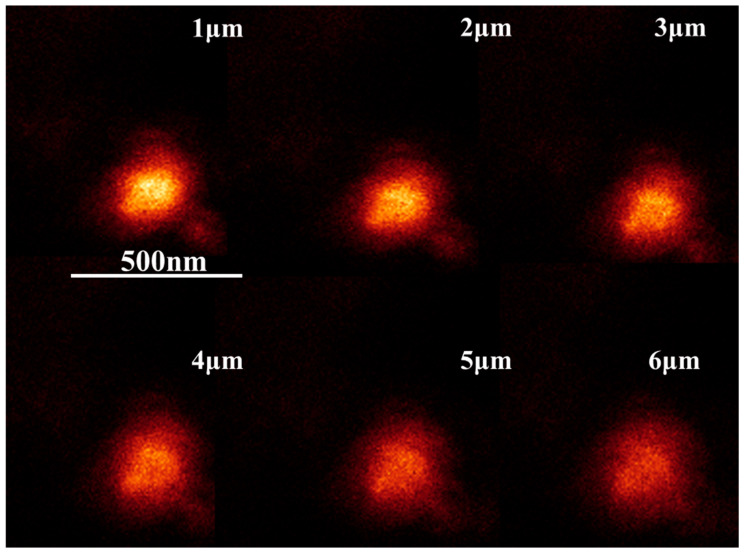
Confocal fluorescence microscopy in Z-scan regime of a selected area of the FCF sensor. λex = 420 nm, Z-plane interval = 1 μm.

Our FCF sensor utilizes changes in spectrally resolved PL signals from CIZS QDs in FCF when brough into contact with aqueous solutions of chosen anions. Since the PL signals were detected using a microspectrophotometer, a uniform distribution of QDs in an FCF cotton matrix was first examined with a laser scanning confocal fluorescence microscope in a Z-scan regime [33] (Figure 6). The bright orange-red fluorescence spots in Figure 6 are from the ensembles of optically excited CIZS QDs, indicating that the QDs are uniformly attached to the cotton fabric and further variations in the PL signal from different areas of the FCF sensor cannot be attributed to the inhomogeneous distribution of QDs over the FCF cotton matrix.

**Figure 7:**
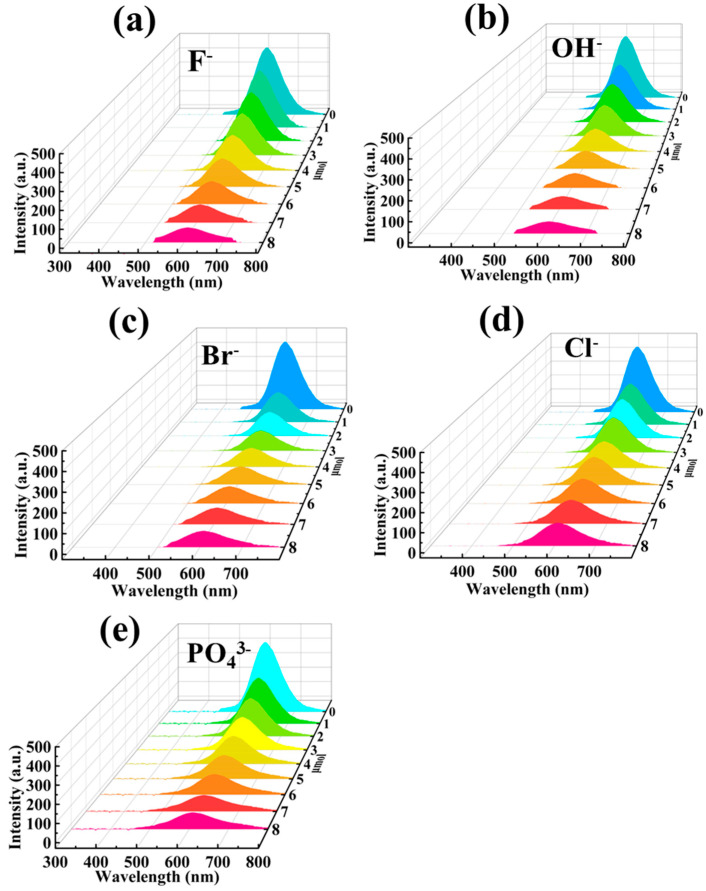
PL spectra of FCF samples after contact with different amounts of PO_4_^3−^ (**a**), Br^−^ (**b**), Cl^−^ (**c**), OH^−^ (**d**), and F^−^ (**e**) anions in aqueous solutions. λex = 420 nm.

Figure 7 shows variations in the PL spectra of the FCF sensor after treatment with different amounts of Br^−^, Cl^−^, F^−^, PO_4_^3−^, and OH^−^ anions in aqueous solutions. We used 0.1 mol/L solutions of the abovementioned anions, but aliquots of different volume are indicated in the corresponding graphs in Figure 7. Figure 7a shows that as the volume of PO_4_^3−^ solution increased from 0 to 8 μmol, the PL signal from the FCF sensor gradually decreased. When the volume reached 10 µmol, the PL signal started to drop significantly, and after 80 μmol, the PL signal was significantly quenched. Figure 7b,e show similar PL quenching after contact with OH^−^ and F^−^ anions in water. Figure 7c,d show the PL spectra from the FCF sensor after treatment with different volumes of Br^−^ and Cl^−^ aqueous solutions. The PL signal from the FCF gradually decreases with increasing amount of anions, which is slightly different from the trend observed after the addition of PO_4_^3−^, OH^−^, and F^−^. Similarly, in Figure 7d, the quenching of fluorescence in the FCS after contact with Cl^−^ is also evident.

The data shown in Figure 7 show that, qualitatively, all the studied anions quench the PL signal from the FCF, but in a slightly different manner. To quantitatively compare their effect, we plotted PL values versus the amount of corresponding anions in the solutions in contact with the FCF. It is important to note that all five anions—Br^−^, Cl^−^, F^−^, PO_4_^3−^, and OH^−^—can be sensed and selected when one of them exists with other anions, except the other four in this group.

**Figure 8:**
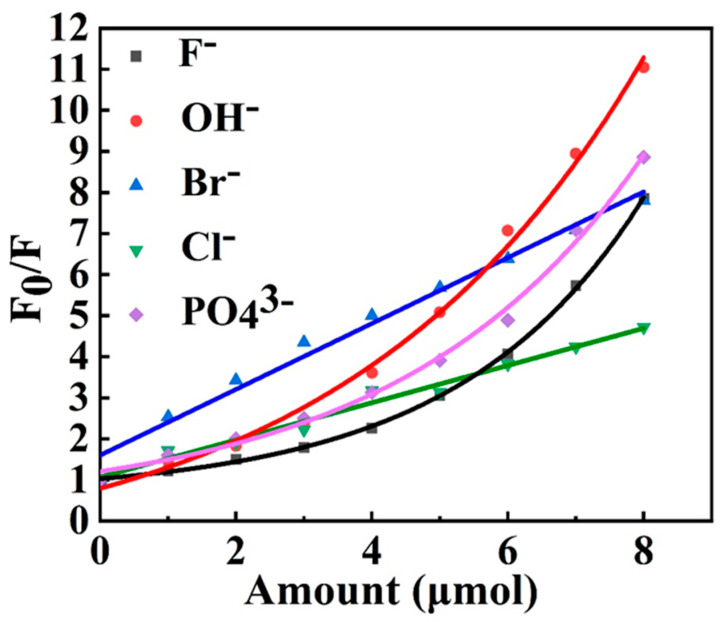
Relative PL quenching curves for FCF after being in contact with Br^−^, Cl^−^, F^−^, PO_4_^3−^, or OH^−^ anions in aqueous solution along with the best math fitting (the fitting parameters are described in the text).

To evaluate the selectivity of the FCS toward these anions, the quenching relationship between the relative PL signal of the FCF and the concentration of each anion was fitted within the amount range of 0–8 µmol, as follows:

F_0_/F = A × (K × C + 1) + B × exp(-C/b_1_) + b_2_ (1)

where F_0_ is the PL intensity of the FCF at the specified emission wavelength in absence, and F is in the presence of selected anions (Figure 8). The quenching of the fluorescence intensity of the FCS by PO_4_^3−^, OH^−^, and F^−^ follows the abovementioned relationship with fitting parameters of R^2^ = 0.9936, R^2^ = 0.9933, and R^2^ = 0.9995, respectively. The effect of Br^−^ and Cl^−^ amount on the relative PL intensity follows a linear relationship with the fitting parameters of R^2^ = 0.9814 and R^2^ = 0.9810, respectively.

The fitting results are shown in the following table.

For the non-linear fitting model, the data in Figure 8 allow us to estimate the detection sensitivity for PO_4_^3−^, OH^−^, Br^−^, Cl^−^, and F^−^ anions. The parameters in Equation 1 for each of the five anions are listed in Table 1, from which one can clearly identify the different anions at the micron molar level. This indicates the high sensitivity of the solid sensor, especially for flexible fabric substrates. Thus, this wearable fabric fluorescence sensor for popular pollutant anions may potentially be an effective tool to detect liquid medium pollution in the environment.

## 4. Conclusions

We have successfully fabricated a novel fluorescent cotton fabric (FCF) sensor grafted with hydrophilic MPA-stabilized Cu-In-Zn-S core–shell QDs. The FCF sensor demonstrates PL response when in contact with aqueous solutions containing various anions, including Br^−^, Cl^−^, F^−^, PO_4_^3−^, and OH^−^. After contact with the abovementioned ions, the PL signal from the FCF remarkably decreased, with linear dependence on the amount of Br^−^ and Cl^−^ and super-linear dependence on the amount of F^−^, PO_4_^3−^, and OH^−^ anions. This preparation method is simple, reproducible, and cost-effective, with broad application prospects. Our results also expand the application of colloidal semiconductor QDs in environmental and biological detection. This work demonstrates an easy and scalable approach to immobilize Cu-In-Zn-S QDs onto cotton fabric, yielding a fluorescent material responsive to multiple anions via photoluminescence quenching. Thus, the current study establishes proof-of-concept under controlled laboratory conditions, setting a solid foundation for future work on real-sample testing in complex matrices (natural waters, wastewater, seawater) using standard addition protocols.

## Figures and Tables

**Table 1:** Fitting data of FCS quenching by five anions.

	A	K	B	b1	b2	R^2^
F^−^	0	0	0.381	−2.720	0.647	0.9995
OH^−^	0	0	2.005	−4.374	−1.213	0.9933
Br^−^	1.600	0.501	0	0	0	0.9814
Cl^−^	1.067	0.425	0	0	0	0.9810
PO_4_^3−^	0	0	0.897	−3.537	0.307	0.9936

## Data Availability

The raw data supporting the conclusions of this article will be made available by the authors on request.
